# Target Values of Cardiovascular Risk Factors Are Not Associated with All-Cause Mortality in Patients with Type 2 Diabetes Mellitus

**DOI:** 10.1371/journal.pone.0124536

**Published:** 2015-04-30

**Authors:** Antonio Pacilli, Olga Lamacchia, Andrea Fontana, Massimiliano Copetti, Mauro Cignarelli, Vincenzo Trischitta, Salvatore De Cosmo

**Affiliations:** 1 Unit of Internal Medicine, Department of Medical Sciences, IRCCS Casa Sollievo della Sofferenza, San Giovanni Rotondo, Italy; 2 Unit of Endocrinology and Metabolic Diseases, Department of Surgical and Medical Sciences, University of Foggia, Italy; 3 Unit of Biostatistics, IRCCS Casa Sollievo della Sofferenza, San Giovanni Rotondo, Italy; 4 Research Unit of Diabetes and Endocrine Diseases, IRCCS Casa Sollievo della Sofferenza, San Giovanni Rotondo, Italy; 5 Department of Experimental Medicine, “Sapienza” University, Rome, Italy; Weill Cornell Medical College Qatar, QATAR

## Abstract

**Background:**

To investigate prospectively the relationship between target values of glycated hemoglobin, blood pressure and LDL-cholesterol, as considered in a combined fashion, and all-cause mortality in patients with type 2 diabetes mellitus.

**Methods:**

Two cohorts of patients with type 2 diabetes mellitus, the Gargano Mortality Study (n=810) and the Foggia Mortality Study (n=929), were investigated. A weighted target risk score was built as a weight linear combination of the recommended targets reached by each patient.

**Results:**

In the Gargano Mortality Study and in the Foggia Mortality Study (mean follow up=7.4 and 5.5 years, respectively), 161 (19.9%) and 220 (23.7%) patients died, with an age and sex adjusted annual incidence rate of 2.1 and 2.8 per 100 person-years, respectively. In both study samples the weighted target risk score tended to be linearly associated with all-cause mortality (HR for one point increment=1.30, 95% CI: 1.11-1.53, p=0.001, and HR=1.08, 95% CI: 0.95-1.24, p=0.243, respectively). When the two cohorts were pooled and analyzed together, a clear association between weighted target risk score and all-cause mortality was observed (HR for one point increment=1.17, 95% CI:1.05-1.30, p=0.004). This counterintuitive association was no longer observable in a model including age, sex, body mass index, smoking habit, estimated glomerular filtration rate, albuminuria and anti-diabetic, anti-hypertensive and anti-dyslipidemic treatment as covariates (HR for one point increment=0.99, 95% CI: 0.87-1.12, p=0.852).

**Conclusions:**

In a real life clinical set of patients with type 2 diabetes mellitus, the combination of recommended target values of established cardiovascular risk factors is not associated with all-cause mortality.

## Introduction

Type 2 diabetes mellitus is a major global health problem affecting an estimated 371 million people worldwide [1]. Compared with people without diabetes, patients with diabetes are at increased mortality risk [2]. In order to limit such a risk, clinicians carry out appropriate multifactorial therapeutic strategies, targeting factors (mainly of cardiovascular origin) which are responsible for mortality excess.

In diabetic patients the target glycated hemoglobin (HbA_1c_) level recommended is 7% (53 mmol/mol) or less, though the need of an individualized approach has been recently stressed by the most updated guidelines [3]. Of note, the results of randomised clinical trials on the benefits on cardiovascular disease of targeting intensive glycemic control in type 2 diabetes mellitus are controversial [4–6]. In addition, meta-analyses of all such trials addressing the impact of intensive glucose lowering therapy on all-cause mortality showed no benefit at all [7–10].

Beyond glycemic control, diabetes care also requires appropriate blood pressure (BP) and LDL-cholesterol (LDL-C) lowering therapies [3].

Aggressive BP reduction in type 2 diabetes mellitus has recently been questioned [3, 11, 12] so that BP treatment goal in diabetic patients is now 140/80 mmHg, with lower targets suggested only for some subgroups, including patients with kidney disease [3].

Finally, LDL-C target levels in diabetic patients are recommended to as less than 100 mg/dl [3], although this may not be beneficial for non-vascular mortality [13] as well as for specific subgroups of patients [14], including those undergoing hemodialysis [15].

To add uncertainty on the role of HbA1c, blood pressure and LDL-C recommended cut-off values on all-cause mortality in patients with type 2 diabetes, multifactorial intervention aimed at promoting an overall intensive management have given conflicting results in terms of reduction of all-cause mortality [16, 17], while a very recent retrospective Chinese study reported no beneficial effect of reaching such recommended cut-off values [18].

Aim of our study was to investigate prospectively in real life clinical set the relationship between target values of HbA_1c_, BP and LDL-C in a combined fashion and all-cause mortality in patients with type 2 diabetes mellitus.

## Materials and Methods

### Ethics Statement

The study and the informed consent procedures were approved by the local Institutional Ethic Committee IRCCS (Istituto di Ricovero e Cura a Carattere Scientifico) ‘‘Casa Sollievo della Sofferenza” and performed according to the Helsinki Declaration. All participants gave written consent.

### Patients

We studied prospectively two previously described [19] cohorts of patients with type 2 diabetes mellitus (according to ADA 2003 criteria). The patients were recruited at the outpatient clinics of our research-based Hospitals, they were clinically stable and not admitted for acute events in the last eight months.

### The Gargano Mortality Study (GMS)

One-thousand-twenty-eight Whites from Italy with type 2 diabetes mellitus were consecutively recruited at Scientific Institute “Casa Sollievo della Sofferenza” in San Giovanni Rotondo (Apulia, Central-Southern Italy) for a study aimed at unraveling predictors of incident all-cause mortality. The only exclusion criterion was the presence of poor life expectancy due to malignancies. Up to date, this cohort has been followed-up for 7.40±2.15 years (range: 0.04–9.83) with the last information on vital status being obtained on November 30^th^ 2010. After excluding patients *(i)* whose information on vital status at follow-up was not available (n = 190) and *(ii)* who had missing data at baseline (n = 28), 810 patients (78.8% of the initial cohort) constituted the eligible sample for the present investigation.

### The Foggia Mortality Study (FMS)

One-thousand-one-hundred-two Whites from Italy with type 2 diabetes mellitus were consecutively recruited at Endocrine Unit of University of Foggia (Apulia, Central-Southern Italy) from January 7^th^ 2002 to September 30^th^ 2008, for a study aimed at unraveling predictors of incident all-cause mortality. Also in this case the only exclusion criterion was the presence of poor life expectancy due to malignancies. Up to date, this cohort has been followed-up for 5.52±2.04 years (range: 0.03–10.83) with the last information on vital status being obtained on March 31^st^ 2013. After excluding patients *(i)* whose information on vital status at follow-up was not available (n = 101) and *(ii)* who had missing data at baseline (n = 72), 929 patients (84.3% of the initial cohort) constituted the eligible sample for the present analysis.

### Data collection

At baseline all patients were interviewed regarding age at diabetes diagnosis, smoke habit and ongoing anti-diabetes, anti-dyslipidemia and anti-hypertension treatments. Duration of diabetes was calculated from the calendar year of data collection minus the calendar year of diabetes diagnosis. All subjects enrolled in the study underwent physical examination, including measurements of height, weight, body mass index (BMI) and BP (i.e., two measurements rounded to the nearest 2 mmHg in the sitting position after at least 5 min rest, using an appropriate-sized cuff; diastolic BP was recorded at the disappearance of Korotokoff sound, phase V). Fasting venous blood was sampled from an antecubital vein from all patients for the measurement of standardized serum creatinine by using the modified kinetic Jaffè reaction (Hitachi 737 Autoanalyzer), total serum cholesterol (enzymatic method, Cobas; Roche Diagnostics, Welwin Garden City, U.K.), HDL-cholesterol, serum triglycerides (enzymatic method, Cobas) and HbA_1c_ (HPLC Diamat Analyzer; Bio-Rad, Richmond, CA); LDL-C was then calculated by the Friedewald formula. Urinary albumin and creatinine concentrations were determined the same morning of the clinical examination from an early-morning first void sterile urine sample by the nephelometric method (Behring Nephelometer Analyzer; Behring, Marburg, Germany) and the Jaffè reaction-rate method, respectively. The urinary ACR was then calculated. Glomerular filtration rate was estimated (e-GFR) by Epidemiology Chronic Kidney Disease formula [20].

### Study endpoint

All-cause mortality was the only pre-specified end point of this study. At follow-up, the vital status of study patients was ascertained by two authors for each study, either by telephone interview with the patient or his/her relatives or by queries to the registry office of cities of residence.

### Statistical analysis

Patients’ baseline characteristics were reported as mean ± standard deviation (SD) and frequency (percentage) for continuous and categorical variables, respectively; overall death incidence rates were calculated as number of deaths per 100 person-years. Age and sex adjusted mortality rates were derived from Poisson regression models, using age and sex as covariates and logarithm of follow-up time as the offset variable. A target score was built as the simple sum of the three domains. Domains were indicator variables which equal to one in case of the presence of the following conditions: HbA_1c_ <7% (53 mmol/mol); systolic BP <140 mmHg and diastolic BP <80 mmHg; LDL-C <100 mg/dl. Due to the low number of patients reaching 3 targets, the target score was re-categorized into three groups as follows: 0, 1 and ≥2 recommended targets (groups: 0, 1, 2, respectively). Comparisons between patients’ baseline characteristics according to categories of target score were performed for each GMS and FMS separately and p-values for linear trend were derived from Spearman correlation coefficient and Cochran-Mantel-Haenszel statistics for continuous and categorical variables, respectively. As for mortality rates, p-value for linear trend was derived from Poisson regression model using target score as continuous covariate and logarithm of follow-up time as the offset variable. The overall survival was defined as the time between enrollment and death; for subjects who did not experience the end point, survival time was censored at the time of the last available follow-up visit. Time-to-death analyses were performed using multivariate Cox proportional hazards regression models and risks were reported as hazard ratios (HR) along with their 95% confidence intervals. Proportional hazard assumption was evaluated using the global score test for a non-zero slope in a generalized linear regression of the scaled Schoenfeld residuals on functions of time [21]. Univariable and multivariable Cox models were estimated for GMS and FMS samples separately, including the target score as continuous and categorical variable, respectively. Moreover, pooled data analyses were performed in an individual patient data meta-analysis fashion [22] (i.e. adjusting for “study sample”) after excluding the presence of heterogeneity. Multivariable models included all relevant variables potentially affecting the effect of target score on mortality risk, such as age, sex, BMI, smoking habit, e-GFR, albuminuria, anti-diabetes treatment, anti-hypertension treatment, anti-dyslipidemia treatment. In addition, a weighted version of the target score was built as a weighted linear combination of all three domains using a study-sample adjusted regression coefficients estimated from a multivariable Cox proportional hazard model as weights. Specifically, a raw score was calculated multiplying each domain by its regression coefficient and summing them. Such raw score was then normalized to vary within a range from 0 to 3: subtracting the observed raw minimum value and then dividing such difference by the observed range (minimum to maximum span), and eventually multiplying for 3. Adjusted survival curves were derived from the fully-adjusted multivariable Cox regression, using the direct approach [23].

A p value <0.05 was considered to be statistically significant. All analyses were performed using SAS Release 9.3 (SAS Institute, Cary, NC, USA) and R (version 2.15, package: “survival”).

### Power calculation

A Cox regression of the log hazard ratio on the target score (which had a standard deviation of 0.76), based on a sample of 1,739 observations achieved 80% power at a 0.05 significance level to detect a HR of 1.21. The sample size was adjusted since a multiple regression of the target score on the other covariates in the Cox regression is expected to have an R Squared of 0.037. The sample size was further adjusted for an anticipated event rate of 0.22.

## Results

Baseline clinical features of the 810 patients from GMS and the 929 patients from FMS are reported in Table 1.

**Table 1:** Baseline clinical features of 1739 patients with type 2 diabetes from Gargano Mortality Study and Foggia Mortality Study.

	GMS (n = 810)	FMS (n = 929)
**Sex (M/F)**	396/414	452/477
**Age (years)**	62.2 ± 9.7	63.7 ± 11.8
**BMI (kg/m** ^**2**^ **)**	31.0 ± 5.7	30.1 ± 6.4
**Smokers: n (%)**	105 (13.0)	155 (17.1)
**Duration of diabetes (years)**	11.1 ± 9.2	13.1 ± 10.0
**Glycated hemoglobin (%)**	8.6 ± 1.9	9.0 ± 2.1
**Systolic blood pressure (mmHg)**	134.4 ± 16.1	130.2 ± 15.6
**Diastolic blood pressure (mmHg)**	78.4 ± 8.7	76.4 ± 9.0
**LDL-cholesterol (mg/dl)**	120.8 ± 37.7	103.5 ± 38.0
**e-GFR (ml min** ^**-1**^ **1.73 m** ^**-2**^ **)**	75.0 ± 20.3	85.5 ± 34.3
**Albuminuria**		
**Missing values (n)**	31	15
**Normo-albuminuria n (%)**	541 (69.4)	543 (59.4)
**Micro/macro-albuminuria n (%)**	238 (30.6)	371 (40.6)
**Antidiabetic therapy**		
**Diet alone: n (%)**	113 (13.9)	135 (14.6)
**OAD: n (%)**	358 (44.2)	456 (49.1)
**Insulin±OAD: n (%)**	339 (41.8)	337 (36.3)
**Arterial hypertension: n (%)**	693 (85.6)	822 (88.5)
**Dyslipidemia: n (%)**	704 (87.2)	795 (85.6)

Data are reported as mean±standard deviation or frequencies (n) and percentages (%), for continuous and categorical variables, respectively.

GMS: Gargano Mortality Study; FMS: Foggia Mortality Study; BMI: body mass index; LDL: low density lipoprotein; e-GFR: estimated-glomerular filtration rate; OAD: oral antidiabetes drugs.

Patients reaching 0 (group 0), 1 (group 1) and 2 or 3 (group 2) recommended targets in the GMS and FMS are shown in Table 2. Clinical features across the three target groups are also shown in Table 2. In both GMS and FMS, age significantly increased across target groups whereas BMI significantly decreased. As expected, given the criteria utilized to build the target score, also HbA_1c_, BP and LDL-C levels as well as the proportion of hypertension and dyslipidemia were significantly different across groups.

**Table 2:** Baseline clinical features of 1739 patients with type 2 diabetes according to categories of target score.

	GMS				FMS			
	Group 0 (n = 305)	Group 1 (n = 344)	Group 2 (n = 161)	*p-for trend* ^*a*^	Group 0 (n = 259)	Group 1 (n = 380)	Group 2 (n = 290)	*p-for trend* ^*a*^
**Sex (M/F)**	150/155	155/189	91/70	0.277	114/145	190/190	148/142	0.106
**Age (years)**	60.6±9.9	63.2±9.6	63.4±9.2	0.001	62.8±11.2	63.7±11.6	64.6±12.5	0.038
**BMI (kg/m** ^**2**^ **)**	31.7±5.9	30.9±5.8	29.6±5.1	<0.001	30.3±6.6	30.4±6.3	29.5±6.4	0.084
**Smokers: n (%)**	47 (15.4)	43 (12.5)	15 (9.4)	0.058	45 (17.6)	63 (17.2)	47 (16.5)	0.716
**Duration of diabetes (years)**	11.1±8.5	11.4±9.5	10.6±9.5	0.319	13.8±9.7	13.1±10.1	12.2±10.0	0.029
**Glycated hemoglobin (%)**	9.1±1.7	8.6±2.0	7.8±1.9	<0.001	9.6±1.8	9.1±2.1	8.2±2.2	<0.001
**Systolic blood pressure (mmHg)**	140.6±15.6	133.3±14.8	125.0±14.4	<0.001	138.1±15.6	131.0±15.0	122.1±12.2	<0.001
**Diastolic blood pressure (mmHg)**	83.1±6.6	77.1±8.4	72.4±7.9	<0.001	82.7±6.7	76.7±8.5	70.3±7.4	<0.001
**LDL-cholesterol (mg/dl)**	139.9±29.4	119.0±37.3	88.5±28.8	<0.001	134.3±32.2	101.2±35.1	78.9±25.3	<0.001
**e-GFR (ml min** ^**-1**^ **1.73 m** ^**-2**^ **)**	75.6±19.1	73.8±20.0	76.5±23.1	0.820	85.7±29.6	86.0±36.1	84.7±36.0	0.254
**Albuminuria**								
**Missing values (n)**	12	12	7		4	8	3	
**Normo-albuminuria n (%)**	199 (67.9)	231 (69.6)	111 (72.1)	0.367	149 (58.4)	204 (54.8)	190 (66.2)	0.054
**Micro/macro-albuminuria n (%)**	94 (32.1)	101 (30.4)	43 (27.9)		106 (41.6)	168 (45.2)	97 (33.8)	
**Antidiabetic therapy**								
**Diet alone: n (%)**	35 (11.5)	48 (13.9)	30 (18.6)		33 (12.8)	58 (15.3)	44 (15.2)	
**OAD: n (%)**	148 (48.5)	139 (40.4)	71 (44.1)	0.248	136 (52.7)	177 (46.6)	143 (49.3)	0.803
**Insulin±OAD: n (%)**	122 (40.0)	157 (45.6)	60 (37.3)		89 (34.5)	145 (38.2)	103 (35.5)	
**Arterial hypertension: n (%)**	275 (90.1)	290 (84.3)	128 (79.5)	<0.001	259 (100.0)	337 (88.7)	226 (77.9)	<0.001
**Dyslipidemia: n (%)**	277 (91.4)	293 (85.4)	134 (83.2)	0.006	239 (92.3)	327 (86.0)	229 (79.0)	<0.001
**Mortality incidence rate (per 100 py)**	2.2	3.0	3.0	0.092 ^b^	4.0	5.7	5.4	0.056 ^b^

Data are reported as mean±standard deviation or frequencies (n) and percentages (%), for continuous and categorical variables, respectively.GMS: Gargano Mortality Study; FMS: Foggia Mortality Study; Group 0: no variable below target levels; Group 1: one variable below target levels; Group 2: 2 or all 3 variables below target levels; BMI, body mass index; LDL, low density lipoprotein; e-GFR: estimated-glomerular filtration rate; OAD: oral antidiabetes drugs.

^**a**^ p-for trend was referred to Speraman correlation coefficient and Cochran-Mantel-Haenszel statistic for continuous and categorical variables, respectively.

^b^ p-for trend was derived from Poisson regression model.

In GMS (mean follow-up = 7.4 years) and in FMS (mean follow-up = 5.5 years), 161 (19.9%) and 220 (23.7%) patients died. Age and sex adjusted annual incidence rates were 2.1 and 2.8 per 100 person-years in GMS and in FMS, respectively.

In both GMS and FMS the target score tended to be linearly associated with all-cause mortality (Table 3).

**Table 3:** Hazard ratios for all-cause mortality of target scores in patients with type 2 diabetes of GMS, FMS and whole sample.

	Category	GMS		FMS			Whole sample			
		HR (95%CI)	*p*	HR (95%CI)	*p*	*p-for interaction* ^†^	HR (95%CI) *	*p* *	HR (95%CI) **	*p* **
**Target risk score**	#	1.20 (0.97–1.47)	0.089	1.18 (0.99–1.41)	0.059	0.945	1.19 (1.04–1.36)	0.010	1.02 (0.86–1.20)	0.825
	2 vs. 0	1.39 (0.90–2.14)	0.138	1.36 (0.98–1.91)	0.070	0.192	1.42 (1.09–1.85)	0.010	1.03 (0.75–1.41)	0.876
	1 vs. 0	1.40 (0.98–2.00)	0.064	0.94 (0.67–1.32)	0.720		1.14 (0.89–1.45)	0.308	0.78 (0.57–1.07)	0.122
**Weighted target risk score**	#	1.30 (1.11–1.53)	0.001	1.08 (0.95–1.24)	0.243	0.090	1.17 (1.05–1.30)	0.004	0.99 (0.87–1.12)	0.852

GMS: Gargano Mortality Study; FMS: Foggia Mortality Study; HR: Hazard Ratios, along with their 95% confidence interval (95%CI).

^#^ HR referred to one point increment.

^†^ Testing whether the association between target score and mortality was differential between GMS and FMS samples.

* Analyses were adjusted for study sample.

** Analyses were adjusted for study sample and for age, sex (M/F), BMI, e-GFR, albuminuria (micro or macro-albuminuria/normo-albuminuria), smoking habits (smokers and ex-smokers/non-smokers), antidiabetes treatment (insulin±OAD/OAD/diet only), anti-hypertension treatment (yes/no), anti-dyslypidemia treatment (yes/no).

**Fig 1:**
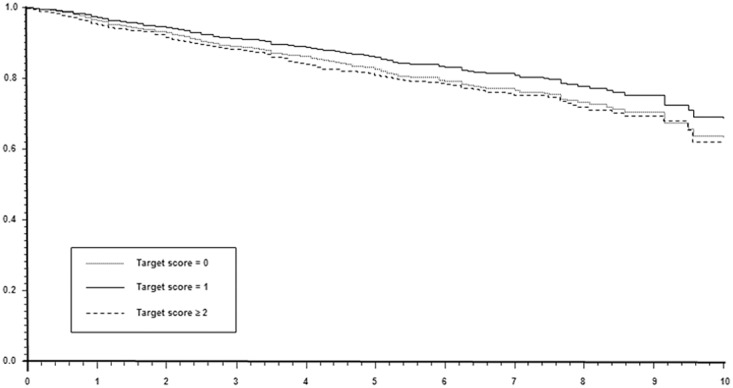
Survival adjusted curves from fully-adjusted multivariable Cox regression, according to the various groups of target attainment.

Given no statistical evidence for heterogeneity (i.e., p for interaction between study sample and target score on all-cause mortality equal to 0.945), the two studies were pooled and analyzed together, in order to increase statistical power. The global score test, based on scaled Shoenfeld residuals for the overall fitting of Cox models, resulted not statistically significant from both sample-adjusted (Chi-Square of 6.293, p = 0.098) and fully-adjusted models (Chi-Square of 13.70, p = 0.473). In the sample-adjusted model, a clear association between target score and all-cause mortality was observed (see Table 3: HR = 1.19, 95% CI: 1.04–1.36, p = 0.010). This association was no longer confirmed in the fully adjusted model (see Table 3: HR = 1.02, 95% CI: 0.86–1.20, p = 0.825), comprising also several covariates (see Statistical Analysis Section), which are known modulators of all-cause mortality risk. Graphical representation of survival curves from such fully-adjusted model according to the various groups of target attainment is reported in Fig 1. In addition, no effect on the association was observed in the subgroups defined by sex (male, n = 848; female, n = 891; p for target risk score-by-sex interaction = 0.37) and age (<65 years, n = 944; ≥65 years, n = 795; p for target risk score-by-age strata interaction = 0.417).

Moreover, when the weighted target risk score was utilized, a tendency toward a linear association with all-cause mortality was observed (HR for one point increment = 1.17, 95% CI:1.05–1.30, p = 0.004, and HR = 0.99, 95% CI: 0.87–1.12, p = 0.852, for sample-adjusted and fully-adjusted model, respectively).

Finally, no statistical association with all-cause mortality was observed in the pooled sample for HbA1c, systolic BP, diastolic BP and LDL-C, when considered in the sample-adjusted analysis (S1 Table).

## Discussion

While several observational and intervention studies in patients with type 2 diabetes mellitus have reported the beneficial effect of targeting traditional cardiovascular risk factors in protecting from micro- and macro-vascular events, the reported effect on all-cause mortality has been conflicting [16, 17, 24–40].

To deeper explore this issue we tested prospectively the association between target levels of HbA_1c_, BP and LDL-C and all-cause mortality in a prospective observational study. To test the combined effect of the three target levels, a weighted risk score was built, according to the number and the individual weight of recommended targets reached by each patient. Quite unexpectedly, we observed a progressively increased risk of all cause mortality paralleling a progressive increase in the number of reached targets; such counterintuitive association was no longer significant after adjusting for possible confounders (including age, sex, BMI, smoking habit, e-GFR and albuminuria); in this fully adjusted model, HR approached one, thus strongly suggesting a neutral effect on mortality risk of targeting major cardiovascular risk factors. Some caution is, nonetheless, needed in interpreting such data because, due to a less than optimal study statistical power, we cannot entirely exclude a false negative result. In this context, it is worth noting that our findings are along the same line of those from a multicenter, cluster randomized trial, the Anglo-Danish-Dutch Study of Intensive Treatment In People with Screen Detected Diabetes in Primary Care (ADDITION-Europe) [16]. In this study the authors investigated in a large cohort of patients with type 2 diabetes mellitus the effect of early multifactorial treatment on cardiovascular events and death; after a 5.3 years follow-up, the intervention had no significant impact on the incidence of both cardiovascular events and all-cause mortality. Of note, the authors found no interaction between intensive treatment and age of study subjects as we also did. More recent information comes from the Action for Health in Diabetes (Look AHEAD) Study [25]. In this trial the intensive lifestyle intervention, although ameliorating some cardiovascular risk factors, did not significantly reduce the rate of death either of cardiovascular or of any cause. Similarly, in a large cohort of patients with type 1 diabetes mellitus, no significant impact on all-cause mortality was observed by achieving HbA_1c_, BP and LDL-C recommended targets [26].

Conversely, intensive multifactorial treatment in a small cohort of patients (n = 160) with longstanding type 2 diabetes mellitus and microalbuminuria was associated with reduced all-cause mortality [17].

Although not our major aim, we also observed no association between individual risk factors as singly considered and all-cause mortality. Also the role of intensive treatment of single targets on all-cause mortality is under debate. While a number of observational studies have shown a significant association between HbA_1c_ levels and all-cause mortality [27–29], intervention trials failed to confirm this link [30–32].

Similarly, the role of reaching stringent systolic (i.e. <130 mmHg) and diastolic (i.e. <75 mmHg) blood pressure level is also questioned in people with diabetes [3, 33–35].

Regarding LDL-C, although levels below 100 mg/dl or, in very high-risk patients, below 70 mg/dl is strongly suggested by The National Cholesterol Education Program Adult Treatment Panel III Guidelines, the beneficial effects of such aggressive LDL-C level cut-offs on all-cause mortality are conflicting both in diabetic patients [36] and in the general population [37, 38]. In this context, it is of note that several epidemiological studies have raises doubts on the relevance of elevated cholesterol levels as important risk factor for all-cause mortality, particularly among elderly people [39, 40], like many diabetic patients are. Finally, no evidence from randomized controlled trials to support continued use of specific LDL-C and/or non-HDL-cholesterol treatment targets is presently available [41], thus calling for cautious when addressing this issue.

Our data are in accord with those from Chiang et al. who have recently shown as the lowest rate of all-cause mortality occurs among patients with HbA1c, SBP and LDL-C in the range of 7.0–8.0%, 130–140 mmHg, 100–130 mg/dl, respectively [18]. Notably, two of such cut-offs values (i.e. those referring to HbA1c and LDL-C) are above those recommended by all current clinical guidelines [3].

Unraveling the mechanisms that underline the lack of association we observed is beyond the scope of our study and we can here only offer some speculations. For example, we can’t exclude the role of non-traditional risk factors (i.e. insulin resistance, homocysteine, low-grade chronic inflammation), which could have obscured the beneficial effect of recommended targets of traditional risk factors [42].

Given the intrinsic nature of our prospective study design (i.e, observational rather than interventional), we cannot exclude that patients who at baseline had a more severe form of diabetes (and therefore a higher mortality risk) were treated more aggressively. Such a bias may have well obscured the beneficial effect of multifactorial treatment on mortality rate.

Finally, among patients with HbA_1c_ below 7% (53 mmol/mol), an increase in hypoglycemic events and eventually mortality rate could also have contributed in neutralizing some beneficial effect of tight glycemic control [43].

The strengths of our study reside mainly in the deep patient phenotype characterization, including the availability of several variables related to cardiovascular risk. Moreover, it is of note that the two samples here analyzed are quite homogenous in terms of clinical features and both environmental and genetic backgrounds, the two Institutions in which recruitment was carried out being only 50 kilometres apart.

Study limitations should be also acknowledged. Our population is hospital based, making uncertain the generalizability of our finding. Second, the availability of only baseline values of the exposures we used, with no information in the following years, might underestimate risk association secondary to a regression dilution during the follow-up period. This limitation applies particularly to pharmacological interventions, which could have been changed during follow-up. An additional limitation is the lack of information on specific causes of mortality, including that of cardiovascular origin. Finally, all BP measurements were manually performed, so that we cannot exclude a measurement bias.

In conclusion, in a real life clinical set of patients with type 2 diabetes mellitus, recommended target values of established cardiovascular risk factors are not associated with reduced all-cause mortality.

## Supporting Information

S1 Table Hazard ratios for all-cause mortality of each single risk factor in patients with type 2 diabetes. HR: Hazard Ratios, along with their 95% confidence interval (95%CI) per standard deviation increment; SD: standard deviation to which HR are referred; OAD: Oral Anti-diabetes Drugs.* Analyses were adjusted for study sample.** Analyses were adjusted for study sample and for age, sex (M/F), BMI, e-GFR, albuminuria (micro or macro-albuminuria/normo-albuminuria), smoking habits (smokers and ex-smokers/non-smokers), antidiabetes treatment (insulin±OAD/OAD/diet only), anti-hypertension treatment (yes/no), anti-dyslypidemia treatment (yes/no).
(DOCX)

## References

[1] IDF Diabetes Atlas Update 2012. Available: http://www.idf.org/diabetesatlas/5e/Update2012.

[2] Emerging Risk Factors Collaboration, Seshasai SR, Kaptoge S, Thompson A, Di Angelantonio E, Gao P, et al Diabetes mellitus, fasting glucose, and risk of cause-specific deaths. N Engl J Med. 2011;364: 829–841. 10.1056/NEJMoa1008862 21366474 PMC4109980

[3] American Diabetes Association. Standards of Medical Care in Diabetes 2014. Diabetes Care. 2014;37(1): S14–80. 24357209 10.2337/dc14-S014

[4] Action to Control Cardiovascular Risk in Diabetes Study Group, Gerstein HC, Miller ME, Byington RP, Goff DC Jr, Bigger JT, et al Effects of intensive glucose lowering in type 2 diabetes. N Engl J Med. 2008;358: 2545–2559. 10.1056/NEJMoa0802743 18539917 PMC4551392

[5] ADVANCE Collaborative Group, Patel A, MacMahon S, Chalmers J, Neal B, Billot L, et al Intensive blood glucose control and vascular outcomes in patients with type 2 diabetes. N Engl J Med. 2008;358: 2560–2572. 10.1056/NEJMoa0802987 18539916

[6] Duckworth W, Abraira C, Moritz T, Reda D, Emanuele N, Reaven PD, et al Glucose control and vascular complications in Veterans with type 2 diabetes. N Engl J Med. 2009;360: 129–139. 10.1056/NEJMoa0808431 19092145

[7] Ray KK, Seshasai SR, Wijesuriya S, Sivakumaran R, Nethercott S, Preiss D, et al Effect of intensive control of glucose on cardiovascular outcomes and death in patients with diabetes mellitus: a meta-analysis of randomised controlled trials. Lancet. 2009;373(9677): 1765–1772. 10.1016/S0140-6736(09)60697-8 19465231

[8] Kelly TN, Bazzano LA, Fonseca VA, Thethi TK, Reynolds K, He J, et al Systematic review: glucose control and cardiovascular disease in type 2 diabetes. Ann Intern Med. 2009;151: 394–403. 19620144 10.7326/0003-4819-151-6-200909150-00137 PMC3058557

[9] Boussageon R, Bejan-Angoulvant T, Saadatian-Elahi M, Lafont S, Bergeonneau C, Kassai B, et al Effect of intensive glucose lowering treatment on all cause mortality, cardiovascular death, and microvascular events in type 2 diabetes: meta-analysis of randomised controlled trials. BMJ 2011;343: d4169 10.1136/bmj.d4169 21791495 PMC3144314

[10] Hemmingsen B, Lund SS, Gluud C, Vaag A, Almdal T, Hemmingsen C, et al Intensive glycaemic control for patients with type 2 diabetes: systematic review with meta-analysis and trial sequential analysis of randomised clinical trials. BMJ. 2011;343: d6898 10.1136/bmj.d6898 22115901 PMC3223424

[11] Vamos EP, Harris M, Millett C, Pape UJ, Khunti K, Curcin V, et al Association of systolic and diastolic blood pressure and all cause mortality in people with newly diagnosed type 2 diabetes: retrospective cohort study. BMJ. 2012;345: e5567 10.1136/bmj.e5567 22936794 PMC3431284

[12] Rönnback M, Isomaa B, Fagerudd J, Forsblom C, Groop PH, Tuomi T, et al Complex Relationship Between Blood Pressure and Mortality in Type 2 Diabetic Patients. Follow-Up of the Botnia Study. Hypertension. 2006;47: 168–173. 16380522 10.1161/01.HYP.0000199667.30253.b7

[13] Cholesterol Treatment Trialists' (CTT) Collaborators, Kearney PM, Blackwell L, Collins R, Keech A, Simes J, Peto R et al Efficacy of cholesterol-lowering therapy in 18,686 people with diabetes in 14 randomised trials of statins: a meta-analysis. Lancet. 2008;371(9607): 117–125. 10.1016/S0140-6736(08)60104-X 18191683

[14] Baigent C, Landray MJ, Reith C, Emberson J, Wheeler DC, Tomson C, et al The effects of lowering LDL cholesterol with simvastatin plus ezetimibe in patients with chronic kidney disease (Study of Heart and Renal Protection): a randomised placebo-controlled trial. Lancet. 2011;377(9784): 2181–2192. 10.1016/S0140-6736(11)60739-3 21663949 PMC3145073

[15] Fellström BC, Jardine AG, Schmieder RE, Holdaas H, Bannister K, Beutler J, et al Rosuvastatin and cardiovascular events in patients undergoing hemodialysis. N Engl J Med. 2009;360: 1395–1407. 10.1056/NEJMoa0810177 19332456

[16] Griffin SJ, Borch-Johnsen K, Davies MJ, Khunti K, Rutten GEHM, Sandbaek A, et al Effect of early intensive multifactorial therapy on 5-year cardiovascular outcomes in individuals with type 2 diabetes detected by screening (ADDITION-Europe): a cluster-randomised trial. Lancet. 2011;378: 156–167. 10.1016/S0140-6736(11)60698-3 21705063 PMC3136726

[17] Gæde P, Lund-Andersen H, Parving HH, Pedersen O. Effect of a Multifactorial Intervention on Mortality in Type 2 Diabetes. N Engl J Med. 2008;358: 580–591. 10.1056/NEJMoa0706245 18256393

[18] Chiang H-H, Tseng F-Y, Wang C-Y, Chen C-L, Chen Y-C, See TT, et al All-Cause Mortality in Patients with Type 2 Diabetes in Association with Achieved Hemoglobin A1c, Systolic Blood Pressure, and Low-Density Lipoprotein Cholesterol Levels. PLoS ONE. 2014;9(10): e109501 10.1371/journal.pone.0109501 25347712 PMC4210124

[19] De Cosmo S, Copetti M, Lamacchia O, Fontana A, Massa M, Morini E, et al Development and validation of a predicting model of all-cause mortality in patients with type 2 diabetes. Diabetes Care. 2013;36: 2830–2835. 10.2337/dc12-1906 23637348 PMC3747924

[20] Levey AS, Stevens LA, Schmid CH, Zhang YL, Castro AF, Feldman HI, et al A New Equation to Estimate Glomerular Filtration Rate. Ann Intern Med. 2009;150: 604–612. 19414839 10.7326/0003-4819-150-9-200905050-00006 PMC2763564

[21] Grambsch P, Therneau T. Proportional hazards tests and diagnostics based on weighted residuals. Biometrika. 1994;81, 515–26.

[22] Olkin I, Sampson A. Comparison of meta-analysis versus analysis of variance of individual patient data. Biometrics. 1998;54 (1) 317e322 9544524

[23] Ghali WA, Quan H, Brant R, van Melle G, Norris CM, Faris PD, et al APPROACH Investigators. Comparison of 2 methods for calculating adjusted survival curves from proportional hazards models. JAMA. 2001;286:1494–1497. 11572743 10.1001/jama.286.12.1494

[24] Holman RR, Paul SK, Bethel MA, Matthews DR, Neil HAW. 10-Year Follow-up of Intensive Glucose Control in Type 2 Diabetes. N Engl J Med. 2008;359: 1577–1589. 10.1056/NEJMoa0806470 18784090

[25] The Look AHEAD Research Group, Wing RR, Bolin P, Brancati FL, Bray GA, Clark JM, et al Cardiovascular Effects of Intensive Lifestyle Intervention in Type 2 Diabetes. N Engl J Med. 2013;369: 145–154. 10.1056/NEJMoa1212914 23796131 PMC3791615

[26] Lithovius R, Harjutsalo V, Forsblom C, Saraheimo M, Groop PH. The consequences of failure to achieve targets of guidelines for prevention and treatment of diabetic complications in patients with type 1 diabetes. Acta Diabetol. 2015;52(1):31–8. 10.1007/s00592-014-0595-x 24849006

[27] Khaw KT, Wareham N, Luben R, Bingham S, Oakes S, Welch A, et al Glycated haemoglobin, diabetes, and mortality in men in Norfolk cohort of European Prospective Investigation of Cancer and Nutrition (EPIC—Norfolk). BMJ. 2001;322: 1–6. 11141143 10.1136/bmj.322.7277.15 PMC26599

[28] Currie CJ, Peters JR, Tynan A, Evans M, Heine RJ, Bracco OL, et al Survival as a function of HbA1c in people with type 2 diabetes: a retrospective cohort study. Lancet. 2010;6;375(9713): 481–489. 10.1016/S0140-6736(09)61969-3 20110121

[29] Nicholas J, Charlton J, Dregan A, Gulliford MC. Recent HbA1c Values and Mortality Risk in Type 2 Diabetes. Population-Based Case-Control Study. PLoS ONE. 2013;8(7): e68008 10.1371/journal.pone.0068008 23861845 PMC3702542

[30] Ray KK, Seshasai SR, Wijesuriya S, Sivakumaran R, Nethercott S, Preiss D, et al Effect of intensive control of glucose on cardiovascular outcomes and death in patients with diabetes mellitus: a meta-analysis of randomised controlled trials. Lancet. 2009;373: 1765–1772. 10.1016/S0140-6736(09)60697-8 19465231

[31] Turnbull FM, Abraira C, Anderson RJ, Byington RP, Chalmers JP, Duckworth WC, et al Intensive glucose control and macrovascular outcomes in type 2 diabetes. Diabetologia. 2009;52: 2288–2298. 10.1007/s00125-009-1470-0 19655124

[32] Rémy Boussageon R, Bejan-Angoulvant T, Saadatian-Elahi M, Lafont S, Bergeonneau C, Kassai B, et al Effect of intensive glucose lowering treatment on all cause mortality, cardiovascular death, and microvascular events in type 2 diabetes: meta-analysis of randomised controlled trials. BMJ. 2011;343: d4169 10.1136/bmj.d4169 21791495 PMC3144314

[33] ACCORD Study Group, Cushman WC, Evans GW, Byington RP, Goff DC Jr, Grimm RH Jr, et al Effects of intensive blood-pressure control in type 2 diabetes mellitus. N Engl J Med. 2010;362(17): 1575–1585. 10.1056/NEJMoa1001286 20228401 PMC4123215

[34] Bryg RJ, Graettinger WF. The Hypertension Optimal Treatment Study: What Did It Give Us? Current Hypertension Reports. 1999;1: 337–341. 10981087 10.1007/s11906-999-0043-4

[35] James PA, Oparil S, Carter BL, Cushman WC, Dennison-Himmelfarb C, Handler J, et al Evidence-Based Guideline for the Management of High Blood Pressure in Adults Report From the Panel Members Appointed to the Eighth Joint National Committee (JNC 8). JAMA. 2014;311(5): 507–520. 10.1001/jama.2013.284427 24352797

[36] Colhoun HM, Betteridge DJ, Durrington PN, Hitman GA, Neil HA, Livingstone SJ, et al Primary prevention of cardiovascular disease with atorvastatin in type 2 diabetes in the Collaborative Atorvastatin Diabetes Study (CARDS): multicentre randomised placebo-controlled trial. Lancet. 2004;364(9435): 685–696. 15325833 10.1016/S0140-6736(04)16895-5

[37] Heart Protection Study Collaborative Group. MRC/BHF Heart Protection Study of cholesterol lowering with simvastatin in 20,536 high-risk individuals: a randomised placebo-controlled trial. Lancet. 2002;360: 7–22. 12114036

[38] The ALLHAT Officers and Coordinators for the ALLHAT Collaborative Research Group. Major Outcomes in Moderately Hypercholesterolemic, Hypertensive Patients Randomized to Pravastatin vs Usual Care: The Antihypertensive and Lipid-Lowering Treatment to Prevent Heart Attack Trial (ALLHAT-LLT). JAMA. 2002;288: 2998–3007. 12479764 10.1001/jama.288.23.2998

[39] Schatz IJ, Masaki K, Yano K, Chen R, Rodriguez BL, Curb JD. Cholesterol and all-cause mortality in elderly people from the Honolulu Heart Program: a cohort study. Lancet. 2001;358(9279): 351–5. 11502313 10.1016/S0140-6736(01)05553-2

[40] Iso H, Naito Y, Kitamura A, Sato S, Kiyama M, Takayama Y, et al Serum total cholesterol and mortality in a Japanese population. J Clin Epidemiol. 1994;47: 961–969. 7730912 10.1016/0895-4356(94)90110-4

[41] Stone NJ, Robinson JG, Lichtenstein AH, Bairey Merz CN, Blum CB, Eckel RH, et al ACC/AHA guideline on the treatment of blood cholesterol to reduce atherosclerotic cardiovascular risk in adults: a report of the American College of Cardiology/American Heart Association Task Force on Practice Guidelines. J Am Coll Cardiol. 2014;63(25 Pt B): 2889–2934. 10.1016/j.jacc.2013.11.002 24239923

[42] Goldman N, Turra CM, Glei DA, Seplaki CL, Lin YH, Weinstein M. Predicting mortality from clinical and nonclinical biomarkers. J Gerontol A Biol Sci Med Sci. 2006;61(10): 1070–1074. 17077201 10.1093/gerona/61.10.1070

[43] Zoungas S, Patel A, Chalmers J, de Galan BE, Li Q, Billot L, et al Severe Hypoglycemia and Risks of Vascular Events and Death. N Engl J Med. 2010;363: 1410–1418. 10.1056/NEJMoa1003795 20925543

