# Supplementary material for: Target Values of Cardiovascular Risk Factors Are Not Associated with All-Cause Mortality in Patients with Type 2 Diabetes Mellitus
Source: PLoS One. 2015 Apr 30;10(4):e0124536. doi: 10.1371/journal.pone.0124536 (PMC4415933; doi:10.1371/journal.pone.0124536)
Supplement: S1 Table — HR: Hazard Ratios, along with their 95% confidence interval (95%CI) per standard deviation increment; SD: standard deviation to which HR are referred; OAD: Oral Anti-diabetes Drugs.* Analyses were adjusted for study sample.** Analyses were adjusted for study sample and for age, sex (M/F), BMI, e-GFR, albuminuria (micro or macro-albuminuria/normo-albuminuria), smoking habits (smokers and ex-smokers/non-smokers), antidiabetes treatment (insulin±OAD/OAD/diet only), anti-hypertension treatment (yes/no), anti-dyslypidemia treatment (yes/no). (DOCX) [file pone.0124536.s001.docx]

**S1 Table. Hazard ratios for all-cause mortality of each single risk factor in patients with type 2 diabetes**

|  | **Category** | **HR (95%CI) *** | ***p* *** | **HR (95%CI) **** | ***p* **** |
| --- | --- | --- | --- | --- | --- |
| **Glycated hemoglobin (%)** | (SD: 2.03) | 1.07 (0.97-1.19) | 0.153 | 1.04 (0.91-1.18) | 0.593 |
| **Systolic blood pressure (mmHg)** | (SD: 15.98) | 1.08 (0.98-1.19) | 0.124 | 1.00 (0.88-1.14) | 0.998 |
| **Diastolic blood pressure (mmHg)** | (SD: 8.93) | 0.80 (0.72-0.88) | <0.001 | 1.00 (0.88-1.14) | 0.963 |
| **LDL-cholesterol (mg/dl)** | (SD: 38.87) | 0.81 (0.73-0.90) | <0.001 | 0.95 (0.84-1.08) | 0.476 |
| **Age (years)** | --- | 1.10 (1.09-1.11) | <0.001 |  | |
| **Sex** | M vs. F | 0.96 (0.78-1.17) | 0.673 |  | |
| **BMI (Kg/m^2^)** | --- | 0.97 (0.95-0.98) | <0.001 |  | |
| **e-GFR (ml•min^-1^•1.73 m^-2^)** | (SD: 29.20) | 0.50 (0.45- 0.57) | <0.001 |  | |
| **Albuminuria** | Micro/Macro vs. Normo-albuminuria | 1.80 (1.47-2.21) | <0.001 |  | |
| **Smoking habits** | [Smoker+Ex] vs. No | 0.96 (0.77-1.20) | 0.713 |  | |
| **Anti-diabetes treatment** | Insulin±OAD vs. diet only | 2.09 (1.49-2.94) | <0.001 |  | |
|  | OAD vs. diet only | 0.96 (0.67-1.38) | 0.836 |  | |
| **Anti-hypertension treatment** | Yes vs. No | 2.47 (1.91- 3.19) | <0.001 |  | |
| **Anti-dyslypidemia treatment** | Yes vs. No | 0.77 (0.57-1.03) | 0.073 |  | |
